# 24‐Hour Optimized Lighting for Persons with Dementia: Technology Development

**DOI:** 10.1002/alz.086799

**Published:** 2025-01-09

**Authors:** Jonathon David White, MD. Azaharuddin Ansari, Nafia Al‐Mutawaly

**Affiliations:** ^1^ Yuan Ze University, Taoyuan CIty, Taoyuan Taiwan; ^2^ McMaster University, Hamilton, ON Canada

## Abstract

**Background:**

65% of persons with dementia (PWD) suffer from disturbed sleeping patterns and 28% experience vision related falls. Improved lighting has been shown in numerous studies since the 1980s to mitigate these effects.

**Method:**

Computer code was written to optimize the spectra and intensity of light for vision and non‐vision purposes over a 24‐hour cycle based on off‐the‐shelf LEDs. Hardware was developed to implement the lighting scheme. Feedback was received from practitioners and interested parties.

**Result:**

The 24‐hr dynamic white lighting exceeded the CIE specifications for vision for seniors with high CRI and low Duv at all times. The light’s correlated colour temperature (CCT) and intensity increased rapidly in the morning and dropped slowly into the afternoon and evening. The lighting schedule was implemented using a designated microcontroller that controlled the intensity of four 5‐m LED strips. Three strips were placed in an upper cove near the ceiling and 1 LED strip (nighttime) in woodwork a floor level. The resulting light was uniform throughout the room and shadow‐free. The ratio of horizontal to vertical illuminance was 20% higher than typical direct lighting systems. The final system was certified to Canadian standards and installed in 6 bedrooms at Ressam Gardens Memory Care Community, and at a few other facilities. The majority of feedback about the system was favorable.

**Conclusion:**

A practical modular lighting system that delivers 24‐hr dynamic white lighting has been certified to Canadian standards and implemented in a number of locations in Hamilton, ON Canada serving persons with dementia. The majority of feedback has been positive.

